# Structural and functional characterisation of the methionine adenosyltransferase from *Thermococcus kodakarensis*

**DOI:** 10.1186/1472-6807-13-22

**Published:** 2013-10-18

**Authors:** Julia Schlesier, Jutta Siegrist, Stefan Gerhardt, Annette Erb, Simone Blaesi, Michael Richter, Oliver Einsle, Jennifer N Andexer

**Affiliations:** 1 Institute of Biochemistry, Albert-Ludwigs-University Freiburg, Albertstr. 21, Freiburg D-79104, Germany; 2 Institute of Pharmaceutical Sciences, Albert-Ludwigs-University Freiburg, Albertstr. 25, Freiburg D-79104, Germany; 3 Laboratory for Biomaterials, EMPA - Swiss Federal Laboratories for Materials Science and Technology, Lerchenfeldstrasse 5, CH-9014 St. Gallen, Switzerland; 4 BIOSS Centre for Biological Signalling Studies, Albert-Ludwigs-University Freiburg, Hebelstr. 25, Freiburg D-79104, Germany

**Keywords:** *S*-Adenosylmethionine synthase, *S*-Adenosylmethionine, Thermostable enzyme, Archaea

## Abstract

**Background:**

Methionine adenosyltransferases catalyse the synthesis of *S*-adenosylmethionine, a cofactor abundant in all domains of life. In contrast to the enzymes from bacteria and eukarya that show high sequence similarity, methionine adenosyltransferases from archaea diverge on the amino acid sequence level and only few conserved residues are retained.

**Results:**

We describe the initial characterisation and the crystal structure of the methionine adenosyltransferase from the hyperthermophilic archaeon *Thermococcus kodakarensis.* As described for other archaeal methionine adenosyltransferases the enzyme is a dimer in solution and shows high temperature stability. The overall structure is very similar to that of the bacterial and eukaryotic enzymes described, with some additional features that might add to the stability of the enzyme. Compared to bacterial and eukaryotic structures, the active site architecture is largely conserved, with some variation in the substrate/product-binding residues. A flexible loop that was not fully ordered in previous structures without ligands in the active side is clearly visible and forms a helix that leaves an entrance to the active site open.

**Conclusions:**

The similar three-dimensional structures of archaeal and bacterial or eukaryotic methionine adenosyltransferases support that these enzymes share an early common ancestor from which they evolved independently, explaining the low similarity in their amino acid sequences. Furthermore, methionine adenosyltransferase from *T. kodakarensis* is the first structure without any ligands bound in the active site where the flexible loop covering the entrance to the active site is fully ordered, supporting a mechanism postulated earlier for the methionine adenosyltransferase from *E. coli*. The structure will serve as a starting point for further mechanistic studies and permit the generation of enzyme variants with different characteristics by rational design.

## Background

**Figure 1:**
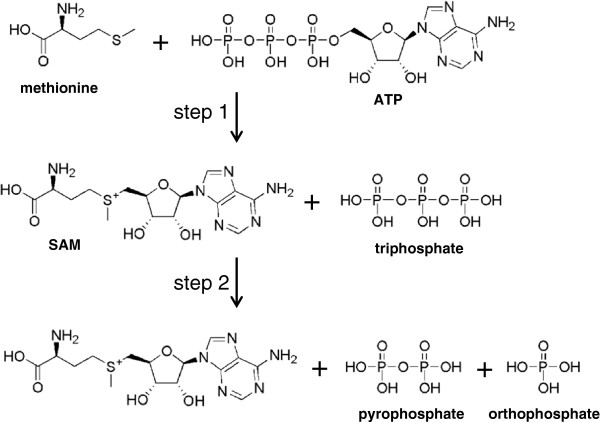
**MAT-catalysed reaction.** In the first step, SAM is formed from ATP and l-methionine; in the second step triphosphate is hydrolysed yielding pyrophosphate and orthophosphate and the products are released.

Methionine adenosyltranferases (*S*-adenosylmethionine synthase, MetK, EC 2.5.1.6, MAT) catalyse the synthesis of *S*-adenosylmethionine (SAM) from adenosine triphosphate (ATP) and methionine. MAT is abundant in all three domains of life, and the cofactor SAM is essential for a variety of different enzymatic reactions. In addition to its function as methyl group donor for C-, N-, S- and O-methylation, SAM participates in the biosynthesis of polyamines and is a source of 5′-deoxyadenosyl radicals that are involved in a broad range of radical-initiated reactions, such as isomerisation, elimination and insertion reactions [1,2]. Recently, an additional function of SAM has been shown, involving dipolar ylide intermediates [3]. As MATs are the only known enzymes catalysing the synthesis of SAM, they are believed to be housekeeping enzymes and have been suggested as tools for phylogenetic analyses [4]. Among the most extensively studied MATs are the bacterial representatives from *Escherichia coli* (*Ec*MAT), [5]*Bacillus subtilis*[6] and several *Streptomyces* species [7], as well as the enzymes derived from selected eukaryotic organisms, such as *Saccharomyces cerevisiae*[8], *Rattus norvegicus*[9], and *Homo sapiens* (*Hs*MAT) [10,11]. MATs catalyse the formation of SAM in a two-step process: in the first step first SAM is formed in a S_N_2 reaction from methionine and the adenosine moiety of ATP; in the second step the triphosphate is cleaved to yield pyrophosphate and orthophosphate and the products are released (Figure 1). Each enzyme dimer has two active sites at the interface of the monomers, and a range of structures with active site ligands such as substrates, products and inhibitors are available. ATP derivatives with non-hydrolysable bonds in the phosphate chain, such as adenosine 5′-(β,γ-imido)triphosphate (AMPPNP), trap the enzyme after the first catalytic step (SAM formation) and have been useful tools in the elucidation of the reaction sequence [12]. Based on *Ec*MAT structures with different ligand combinations a putative mechanism has been suggested, where a conserved histidine acts as an acid to protonate ATP at the O5′-atom (triphosphate-ribose bond), followed by the formation of SAM [13]. Another, substantially different mechanism that involves extensive movements of the substrate ATP in the active site has been proposed for the MAT from *R. norvegicus*[9].

**Figure 2:**
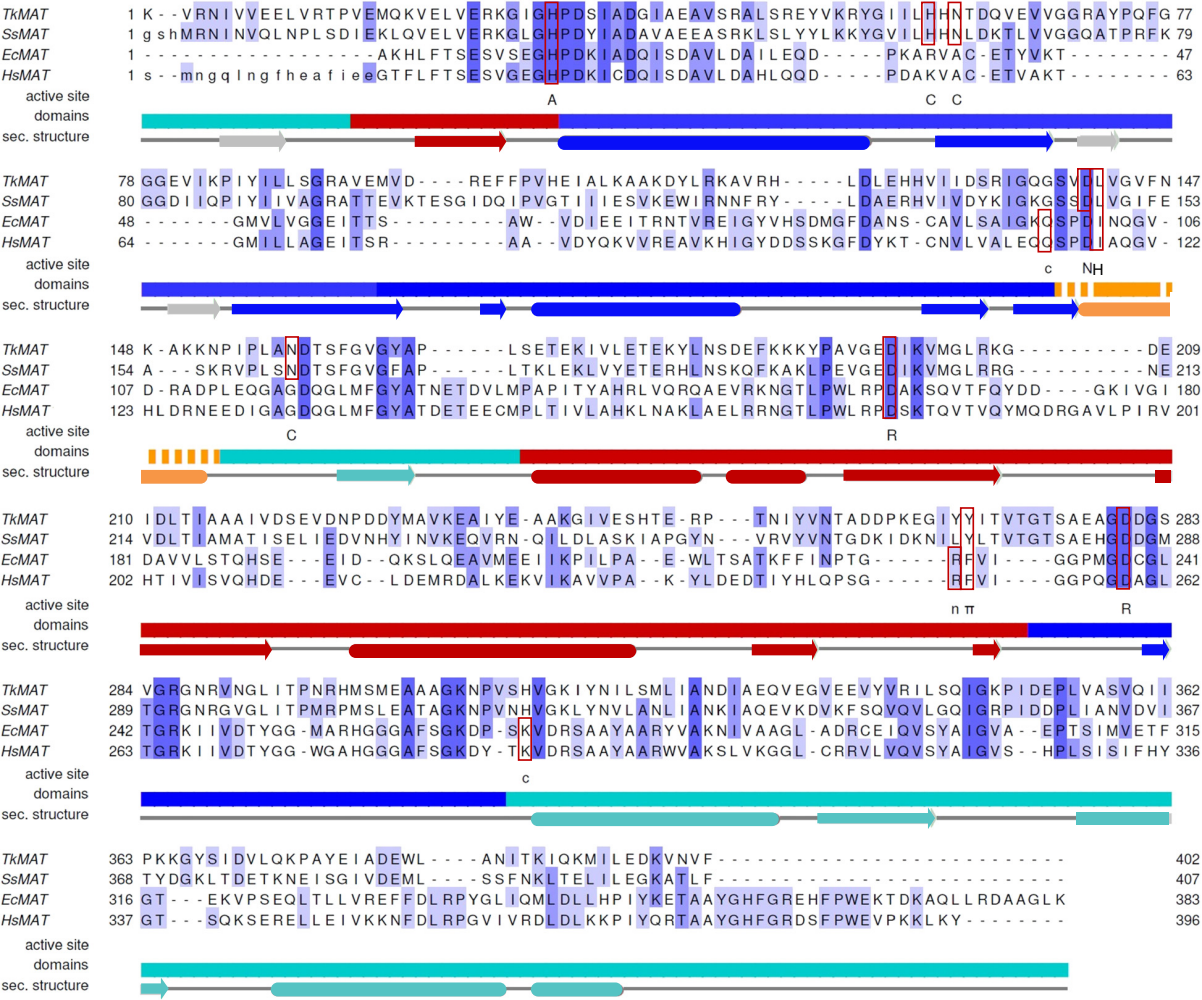
**Structure based sequence alignment of MAT from different domains of life.** Shown are *Tk*MAT, *Ss*MAT (PDB-ID 4HPV), *Ec*MAT (PDB-ID 1RG9) and *Hs*MAT (PDB-ID 2P02). Similar amino acids are highlighted with shades of blue. Active site residues are marked as follows: catalytic histidine (A), π-π-interaction (π), stabilisation of ribose hydroxyls (R), hydrophobic interaction (H), stabilisation of carbonyl (c [*Ec*MAT, *Hs*MAT], C [*Tk*MAT, *Ss*MAT]), interaction with adenine amino group (n [*Ec*MAT, *Hs*MAT], N [*Tk*MAT, *Ss*MAT]). Secondary structure elements for *Tk*MAT are shown under the sequence alignment (rounded rectangles: α-helices; arrows: β-strands; grey β-strands are only present in archaeal MATs). Domains:- N-terminal: red; central: blue; C-terminal: cyan; flexible helix: orange (the extension of the flexible helix differs slightly in the single MATs, the core region is delimited by the unbroken line). The alignment was prepared using STRAP (Interactive Structure based Sequence Alignment Program; http://www.bioinformatics.org/strap/) and annotated using Jalview (http://www.jalview.org).

In contrast to MATs from eukaryotes and bacteria, only two orthologs from archaea have been described [14-16]. After the initial description of an MAT from *Sulfolobus solfataricus* (*Ss*MAT) by Porcelli in 1988 [14], it took more than a decade to identify a *metK* gene encoding MAT in an archaeon due to the distinct phylogeny of these enzymes [16]. Whereas eukaryotic and bacterial MAT share up to 59% of sequence identity (*H. sapiens*/*E. coli* MAT), this figure is around 20% for the archaeal sequences with respect to the other domains of life [15] (Figure 2). Beside their importance from an evolutionary point of view, archaeal enzymes are of great interest for technical applications, mainly due to their origin from extremophilic organisms, often leading to high stability [17,18]. The MAT from *Methanocaldococcus jannaschii* (formerly *Methanococcus jannaschii*) (*Mj*MAT) has been characterised in detail concerning kinetic parameters, optimal reaction conditions, inhibitors and alternative substrates [15,16], as well as for its unfolding behaviour [19,20]. During these studies, a homology model of *Mj*MAT, based on the α-subunit of the human MATII, has been generated, predicting that archaeal MATs exhibit a topology very similar to the eukaryotic and bacterial enzymes [19]. More recently, a crystal structure of *Ss*MAT has become available in the PDB database (PDB-IDs 4HPV, 4K0B), but no associated data has as yet been published.

In the following we present the structural and functional characterisation of MAT from *Thermococcus kodakarensis* (also *T. kodakaraensis*)*,* a new member of the group of archaeal MATs. The sulphur-reducing, hyperthermophilic archaeon *T. kodakarensis* is classified in the phylum euryarchaeota and is found in many high-temperature environments. It grows optimally at a temperature of 85°C and is able to survive up to 100°C [21]. Since 2005, when the genome sequence of *T. kodakarensis* was determined [22], many enzymes involved in several pathways were investigated [23].

## Results

### Optimisation of the HPLC assay

**Figure 3:**
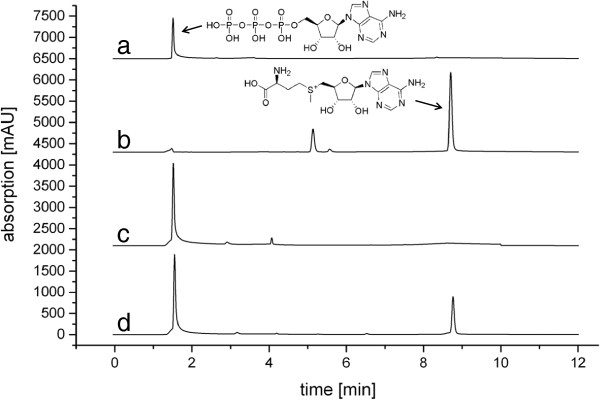
**Activity assay of *Tk*MAT.** Shown are HPLC traces (top to bottom) of: **a)** ATP reference; **b)** SAM reference; **c)** negative control (reaction without enzyme); **d)***Tk*MAT assay. The additional peak in the SAM reference most likely derives from the counter ion present in commercial SAM preparations (*p*-toluenesulfonate salt).

The standard method of determining MAT activity is by using ^14^C-labelled methionine in a cation exchange filter assay. In addition, a few HPLC-based protocols have been described for the direct detection of SAM formed. After some initial tests we decided to use a SCX stationary phase and adapted conditions similar to those described by Kamarthapu *et al.*[6]. A good separation of ATP and SAM could be achieved (Figure 3); the assay is also readily usable in conjunction with detection by mass spectrometry.

### Cloning and initial characterisation of *Tk*MAT

The gene encoding MAT from *T. kodakarensis*[22] was identified by a pBLAST similarity search using the sequence from *M. jannaschii* as the query. Only one gene product was identified (accession number: YP_182958, swissprot: METK_PYRKO), which we cloned from genomic DNA to be heterologously expressed in *E. coli* BL21(DE3)RP. After purification *via* nickel affinity chromatography, SDS-PAGE analysis showed a single band between 40 and 55 kDa corresponding to the calculated mass for a single, his-tagged subunit of 44.3 kDa. In contrast to most eukaryotic and bacterial MATs that form tetramers [24], the archaeal MATs described so far are homodimers in solution. *Tk*MAT follows this trend, as only a single protein peak with a mass of 81 kDa was detected in size exclusion chromatography, corresponding to the homodimer (see Additional file 1). CD denaturation studies resulted in a T_50_ of 85°C (see Additional file 1). The enzyme converted methionine and ATP to SAM (Figure 3), kinetic parameters are summarised in Table 1 (see also Additional file 1).

**Table 1:** Kinetic parameters of MATs from different domains of life

	***Tk*MAT**	***Mj*MAT**	***Ec*MAT**	***Hs*MAT**
**kinetic parameters**				
**V**_**max **_**[μmol/min/mg]**	1.95 ± 0.26	3.0	1.2	0.2
**K**_**M **_**(Met) [mM]**	0.31 ± 0.07	0.14	0.08	0.003
**K**_**M **_**(ATP) [mM]**	6.54 ± 2.49	0.25	0.11	0.03
**assay**	HPLC (SCX column)	radioactive filter binding assay	radioactive filter binding assay	radioactive filter binding assay
**buffer**	0.1 M Tris–HCl, 0.02 M MgCl_2_, 0.2 KCl, pH 8.0	50 mM K-Hepes, 10 mM MgCl_2_, 25 mM KCl, pH 8.0	0.1 Tris-Cl, 6 mM MgCl_2_, 0.1 KCl, pH 8.3	50 mM K-Tes, 50 mM KCl, 10 mM MgCl_2_, 10 mM DTT, 0.3 mM Na_2_EDTA, 0.1 mM BSA, pH 7.4
**substrates**	10 mM l-methionine 10 mM ATP	0.1 mM l-[methyl-^14^C]-methionine 5 mM ATP	0.6 mM l-[methyl-^14^C]-methionine 4.4 mM ^14^C-ATP	0.01 mM l-[methyl-^14^C]-methionine 5 mM ATP
**temperature**	37°C	70°C	25°C	37°C
**reference**	this work	Graham *et al.*[16]	Markham *et al.*[12]	Kotb & Kredich [25]

### Overall structure of *Tk*MAT

**Figure 4:**
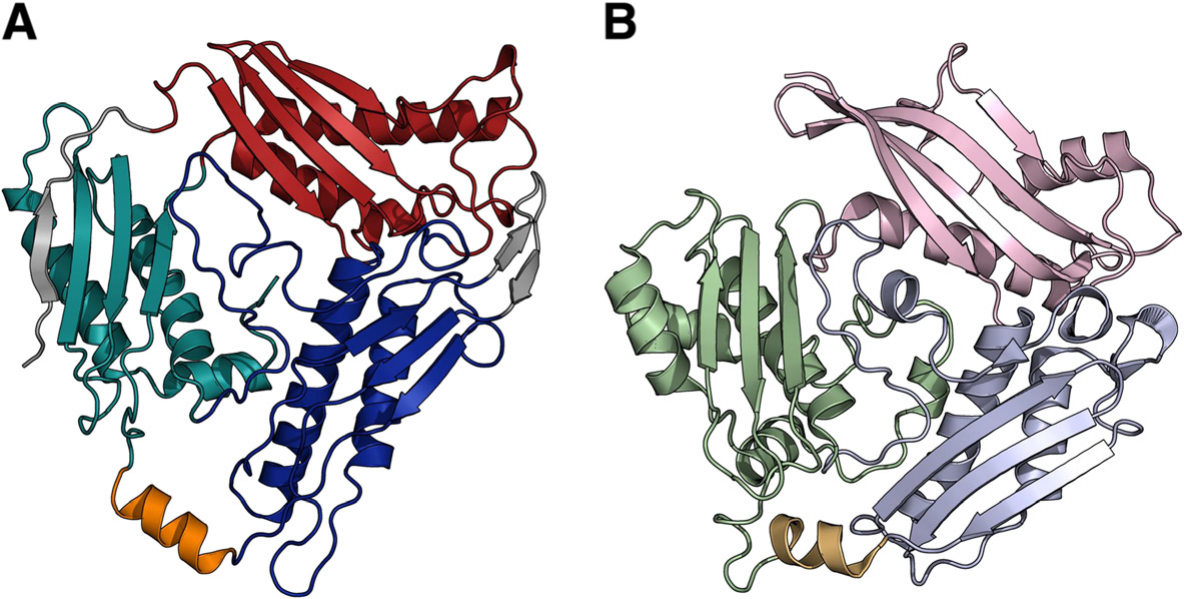
**Domain organisation of *Tk*MAT (A) in comparison with *Ec*MAT (B).***Tk*MAT shows the same general order of sequence stretches participating in the N-terminal (red/ light pink), central (blue/ light blue) and C-terminal (cyan/ light green) domains. The additional β-sheets present in *Tk*MAT are highlighted in grey. The flexible helix covering the active site is shown in orange/ light orange (*Tk*MAT: open conformation; *Ec*MAT: closed conformation).

*Tk*MAT was crystallised by sitting drop vapour diffusion. The crystals belonged to the monoclinic space group *C*2, with four monomers of the enzyme per asymmetric unit. Diffraction data was collected to a maximum resolution of 2.0 Å, and the structure was solved by a combination of SAD using a SeMet derivative of *Tk*MAT and molecular replacement using the *Ss*MAT structure (PDB-ID 4HPV) as a search model. Overall, the structure of *Tk*MAT closely resembles bacterial and eukaryotic MATs (Table 2): the peptide chain folds into three structural – but not topological – domains forming a disc-shaped, trigonal prism. Two such monomers are stacked to form a tightly assembled dimer. *Tk*MAT appears as a dimer in solution, but was found in the crystal packing as a tetramer, which is quite similar to those reported for the MATs from *E. coli*[13] or *R. norvegicus*[9]*.* The three domains of the MAT monomer are commonly described as N-terminal, central and C-terminal domain and consist of non-consecutive stretches of the MAT polypeptide [26]. Compared to bacterial and eukaryotic MATs, *Tk*MAT and *Mj*MAT contain an additional β-strand at the N-terminus (amino acids 1-17 in *Tk*MAT) that forms a part of the C-terminal domain. The other significant structural difference between archaeal MATs and their eukaryotic and bacterial counterparts is an extension of the central two β-strands pleated in the sheet of the central domain (Figure 4).

**Table 2:** r.m.s.d. values for MATs from different domains of life

		***Ss*MAT**	***Ec*MAT**	***Hs*MAT**
		***monomer (A)***	***monomer (A)***	***monomer***
***Tk*MAT**	***monomer (A)***	0.90 Å	8.64 Å	7.85 Å
***Tk*MAT**	***monomer (B)***	1.08 Å	8.55 Å	7.79 Å
		***dimer (A/B)***	***dimer (A/B)***	
***Tk*MAT**	***dimer (A/B)***	1.26 Å	8.05 Å	

### Similarities and differences in the active site

**Figure 5:**
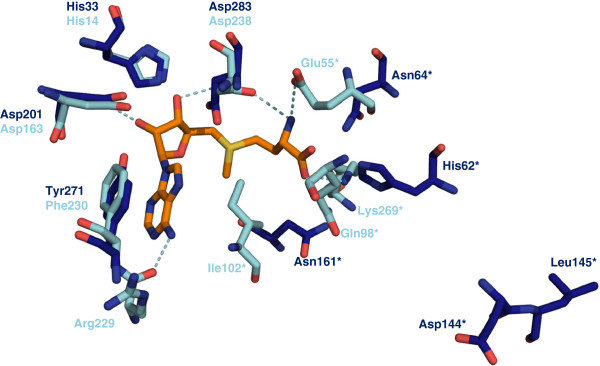
**Active site residues and interaction with SAM.** Shown are the active site residues of *Ec*MAT (light blue; with SAM, PDB entry 1RG9, orange) and the corresponding residues from *Tk*MAT (blue). Most residues stabilising the adenosine moiety are conserved in both structures, whereas the residues interacting with the methionine carboxyl group and the adenine amino group differ. Asp144 and Leu145 (*Tk*MAT) are located on the flexible loop and are therefore not in contact with the substrate in the open position. Amino acids marked with * derive from the second monomer.

The location of the active site can be predicted from the accumulation of water molecules at the dimer interface and is confirmed when superimposed with MAT structures containing ligands such as PDB entries 1RG9 (*Ec*MAT with SAM and [diphosphono]aminophosphonic acid, [13]), 2P02 (*Hs*MAT in complex with SAM, [11]) or 4K0B (*Ss*MAT with SAM and pyrophosphate). Key residues involved in substrate/product stabilisation and catalysis are conserved throughout all available MAT structures (Figures 2 and 5). As described before for MATs we use asterisks (*) to identify amino acids from the second monomer of the dimer. Important examples are: the histidine thought to act as the catalytic acid in SAM formation (His33 in *Tk*MAT, His14 in *Ec*MAT [13]); the aromatic amino acid which is π-stacked with the adenine moiety of ATP/SAM (Tyr271 in *Tk*MAT, Phe230 in *Ec*MAT); the hydrophobic residue interacting with the methionine side chain (Leu145* in *Tk*MAT, Ile102* in *Ec*MAT); and two aspartates coordinating the ribose hydroxyl groups (Asp201/283 in *Tk*MAT, Asp163/238 in *Ec*MAT). Asp283 (*Tk*MAT) and Asp 238 (*Ec*MAT) also coordinate the methionine amino group; in *Ec*MAT another residue (Glu55*) provides additional stabilisation. Other residues conserved in the active sites of bacterial and eukaryotic MATs are not found in the active site of *Tk*MAT. However, the functional residues in archaea are conserved in the two available structures. The most obvious examples for such different stabilising strategies are the hydrogen-bonding networks coordinating the carboxyl group of the methionine moiety, and the adenine amino group. In *Ec*MAT and *Hs*MAT the carboxyl group is stabilised by a glutamine from the central domain (Gln98* in *Ec*MAT, close to the flexible helix described below) and a lysine from the C-terminal domain (Lys269* in *Ec*MAT), whereas in archaeal MATs it interacts with a histidine and an asparagine from the loop region of the central domain (His62*, Asn64* in *Tk*MAT) and another asparagine (Asn161* in *Tk*MAT, C-terminal domain, close to flexible loop). The adenine amino group is stabilised by a hydrogen bond with the carbonyl of an arginine in *Hs*MAT and *Ec*MAT (Arg229, N-terminal domain), as opposed to the side chain carboxyl of an aspartate (flexible loop) of the other monomer in the archaeal structures (Asp144* in *Tk*MAT).

The amino acids involved in the stabilisation of the triphosphate are divers between different MATs. That, and the fact that the ligands bound derive from different steps of the reaction (ATP, triphosphate, (diphosphono)aminophosphonic acid, pyrophosphate, phosphate), makes a comparison imprecise.

### Access to the active site

**Figure 6:**
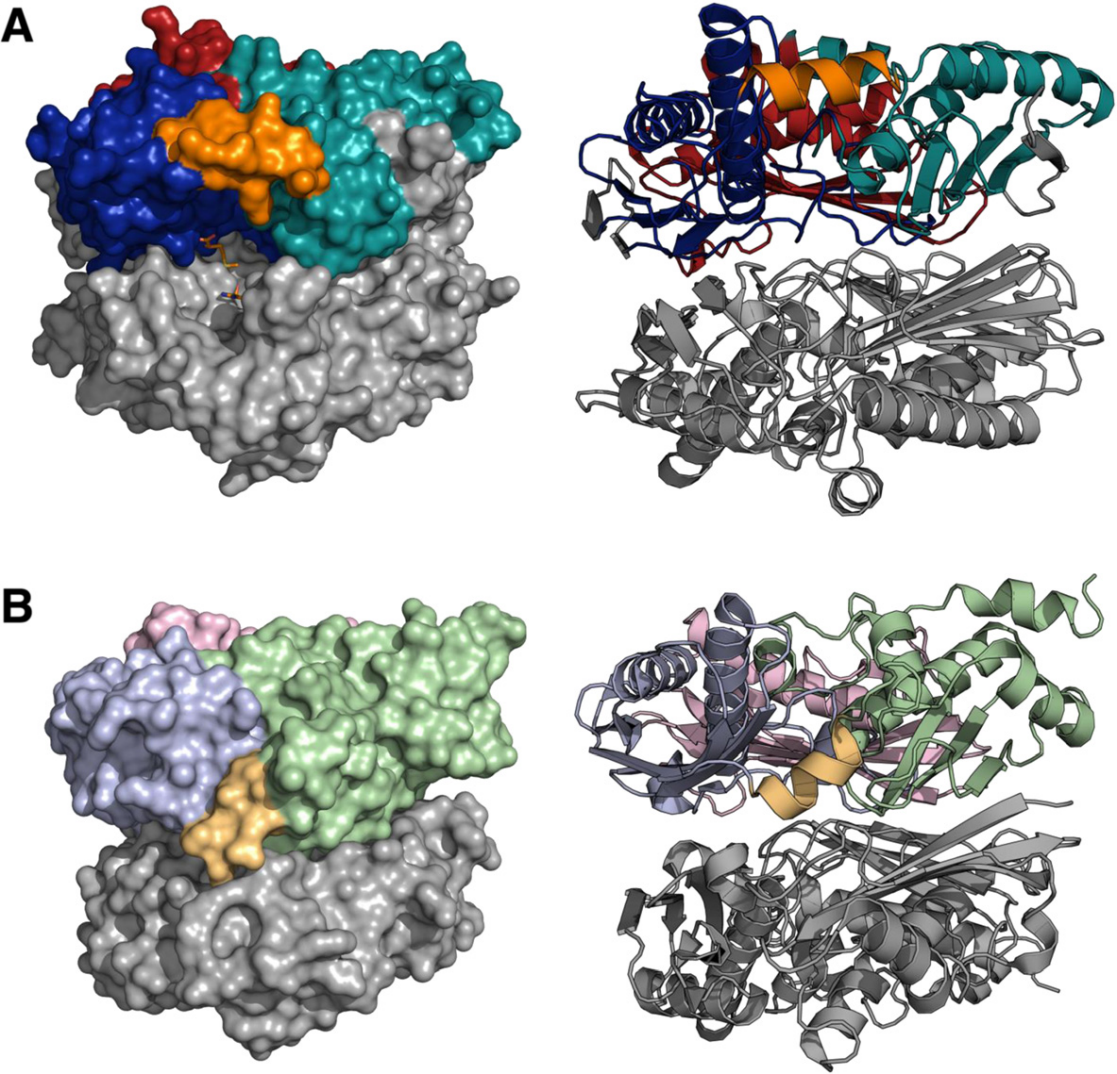
**Open and closed conformations of MAT. A)***Tk*MAT dimer in open conformation (superimposed with SAM from the *E. coli* structure (PDB-ID 1RG9) in the active site [orange] in the surface representation). The domains in the upper subunit are coloured according to Figure 4. **B)***Ec*MAT dimer in closed conformation (PDB-ID 1RG9). See also Additional file 1.

The four monomers of *Tk*MAT within the asymmetric unit are similar in structure, but while the monomers within each homodimer aligned with a root-mean-squared displacement (r.m.s.d.) for all atoms of 0.63 Å (chains A, B) and 0.64 Å (chains C, D), the corresponding monomers in different dimers were nearly identical (r.m.s.d. of 0.14 Å and 0.15 Å). These differences in structure manifest primarily in the β-sheet and the adjacent loops of the C-terminal domain, where a significant asymmetry of the functional dimer of up to 4 Å is present, a similar behaviour can also be observed in other MAT structures. The flexible loop that is suggested to control access to the active site at the dimer interface in MATs [13,27] involves residues 145–155 in *Tk*MAT. In “closed” structures containing bound ligands such as SAM, this part of the protein forms a helix covering the active site (PDB entries 1RG9, 1P7L, 2OBV, 2P02, 4K0B), whereas in most “open” structures the loop is disordered and the active site consequently is accessible [13]. *Tk*MAT is the first structure where the flexible helix is well resolved in all four monomers in an open conformation; it assumes a position close to the outside plane of the monomer and leaves the entrance to the active site open (Figure 6, see Additional file 1).

## Discussion

The high thermostability observed for *Tk*MAT matches the optimal growth temperature of the hyperthermophilic archaeon *T. kodakarensis* very well; similar behaviour was described for the homologous enzymes from *S. solfataricus* and *M. jannaschii*[14,15]. The kinetic parameters obtained are in a similar range to those reported for other MATs, only the K_M_ value for ATP is significantly higher. However, the assays as well as the reaction conditions used in the single experiments, differ widely making a direct comparison difficult (Table 1).

**Figure 7:**
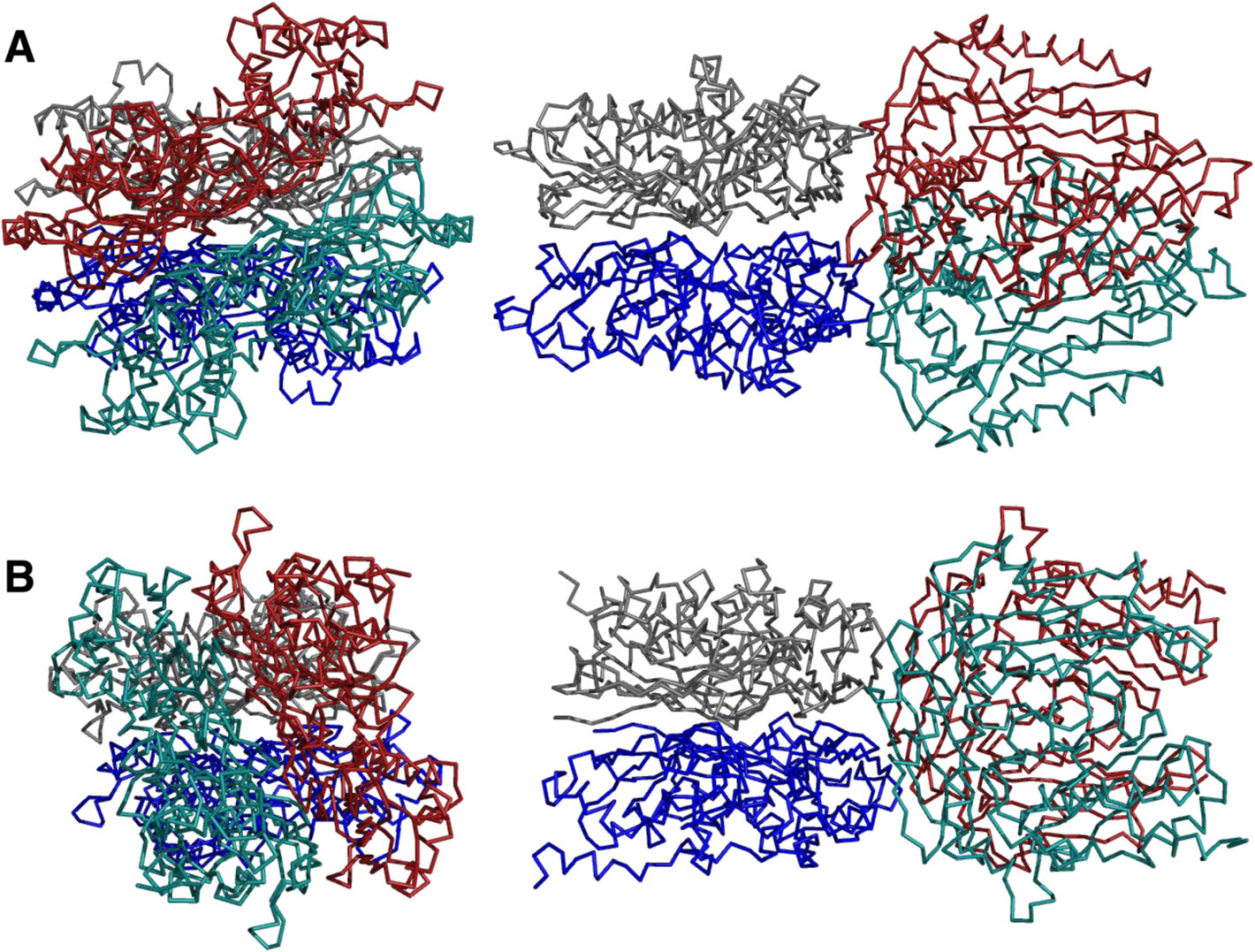
**Spatial arrangement of MAT monomers. A)***Tk*MAT (top and side view): the dimer pairs are twisted in an approximate 30° angle. **B)***Ec*MAT (top and side view): the dimer pairs are stacked at approximately right angle to one another.

The similar overall structures of archaeal and bacterial/eukaryotic MATs suggest that these enzymes share an early common ancestor from which they evolved independently, explaining the low similarity in their amino acid sequences. The tetrameric arrangement found in the crystal occurs in all MATs of known structure, but is frequently only generated through crystallographic symmetry operations. This is in particular the case in eukaryotic and bacterial MATs, where the tetramer shows 222 symmetry that can coincide with crystal symmetry. In contrast, the *Tk*MAT tetramer is generated as a dimer of dimers with a significantly smaller torsion of about 30° (Figure 7). The same arrangement is present in the structure of *Ss*MAT and might be characteristic for archaeal MATs. The only exception from this observation is the structure of the *Burkholderia pseudomallei* MAT (PDB-ID 3IML), with a less pronounced torsion of only 35°.

The additional β-strand in the C-terminal domain is also present in the corresponding *S. solfataricus* structure, but was not represented in the homology model for *Mj*MAT, as this was based on the human structure where the β-strand is absent [19]. However, the first 15 and 16 amino acids of the enzymes from human and rat, respectively, were not ordered in the corresponding crystal structures, so that the presence of the additional β-strand in solution cannot be excluded in these enzymes. In archaeal MATs the additional β-strand extends the β-sheet of the C-terminal domain to match the four-stranded sheets found in the N-terminal and central domains, and therefore might add to the higher stability of archaeal MATs by providing a direct connection between the N- and C-termini of the peptide chain within a stable secondary structure. The extended β-sheet in the central domain might also exhibit stabilisation effects by wrapping around the loops at the edge of the N-terminal domain (see Additional file 1).

A major difference between *Tk*MAT and other MAT structures without any bound ligands is that the helix covering the active site is completely defined in the structure. One exception to this is a structure of *Ec*MAT that was crystallised at low temperatures (PDB-ID 1FUG) [27]. Here, the loop was modelled in a quasi-closed conformation but does not show the ordered helical secondary structure visible in *Ec*MAT structures with bound SAM (PDB-ID 1RG9, 1P7L). Until now the flexible loop was thought to be disordered in the open conformation, however all four monomers in the asymmetric unit of the *Tk*MAT crystal showed the same ordered helix in the open structure. The higher intrinsic stability of the thermophilic enzyme could explain why this region is well defined in the structure of *Tk*MAT. However, in the corresponding open structure from *S. solfataricus* (PDB-ID 4HPV), another thermophilic organism, the loop is not defined.

Our data supports the mechanism of substrate binding described for *Ec*MAT where residues from the flexible loop form their respective interactions with the substrates after they have entered the active site along with movement of the flexible helix into the closed position [13]. The position of these residues (Leu145 [*Tk*MAT], Ile 102 [*Ec*MAT] stabilising the methionine side chain, and Asp144 [*Tk*MAT], Arg229 [*Ec*MAT] interacting with the adenine amino group) are clearly different in the open and closed structures (Figure 5). Some of the active site residues responsible for substrate/product binding in *Ec*MAT and other bacterial and eukaryotic MATs, including the putatively catalytic histidine, are conserved in *Tk*MAT, while some functional groups appear to be stabilised in a different manner. A closer inspection of the active site based on co-crystallised substrates, products or inhibitors will show if these differences are responsible for the variation in the substrate range described for the archaeal MAT from *M. jannaschii,* in comparison to *Ec*MAT [15].

## Conclusions

We present the structure of a thermophilic, archaeal MAT that displays several novel features in comparison to MATs from bacteria or eukarya, including extended β-sheets that may be responsible for the increased stability of MATs from thermophilic organisms. SAM is an important cofactor for a wide range of enzymatic reactions. Enzymes from thermophilic organisms are often used as biocatalysts for technical applications due to their high stability [18]. Commercially available SAM is usually extracted from yeast, however for some applications such as the generation of SAM-derivatives or isotope-labelled compounds using isolated enzymes might be advantageous. The structure described here will serve as a basis for the rational design of MAT variants to further extend the substrate range.

## Methods

### Cloning of *Tk*MAT

The 1155 bp gene of the MAT was amplified by PCR from genomic DNA of *T. kodakarensis* using the following primers: 5′-TATATATACATATGGCAAAACACCTTTTTACGTCCG-3′ and 5′-TATACTCGAGTTACTTCAGACCGGCAGCAT-3′. After restriction with the appropriate restriction enzymes (*Nco*I and *Xho*I, respectively), the fragments were ligated into the pET28a(+) vector finally coding for the MAT carrying an N-terminal His-tag, and *E. coli* BL21(DE3)-CodonPlus-RP competent cells were transformed with the construct.

### Expression and purification of *Tk*MAT

Cells were grown in 500 mL LB Lennox medium supplemented with 34 mg/L chloramphenicol and 50 mg/L kanamycin. Expression was induced by addition of isopropyl thiogalactoside (IPTG, final concentration 0.2 mM) at an optical density (OD_600_) of 0.6. After incubation (4 h, 180 rpm, 37°C) the cells were harvested, resuspended in purification buffer (40 mM Tris-HCl, 100 mM NaCl, pH 8.0) and disrupted by one passage through an EmulsiFlex-B30 homogenizer (Avestin). The enzyme was purified *via* Ni-NTA affinity chromatography. After washing with purification buffer containing 0 and 100 mM imidazole, *Tk*MAT was eluted from the column with with 500 mM imidazole in purification buffer. The protein was desalted on PD-10 columns.

### SeMet-*Tk*MAT

The heterologous expression of the SeMet derivative of *Tk*MAT was performed as described by Dias *et al.*[28]. A preculture of *E. coli* BL21(DE3)RP/pET28a-TkMAT in minimal medium (6 g Na_2_HPO_4_, 3 g KH_2_PO_4_, 1 g NH_4_Cl, 0.5 g NaCl, 1 mM MgSO_4_, 4 g glucose and 0.5 mg thiamine per 980 mL, supplemented with with each 50 mg/L kanamycin and chloramphenicol) was grown for 14 h at 37°C and 180 rpm. The main culture (minimal medium, 500 mL) was incubated at 37°C and 250 rpm. At an OD_600_ of 0.325 an amino acid mix (0.1 g l-lysine, 0.1 g l-phenylalanine, 0.1 g l-threonine, 50 mg l-leucine, 50 mg l-isoleucine, 50 mg l-valine and 50 mg l-selenomethionine in 10 mL 0.5 M HCl, sterile filtrated) and a corresponding amount of NaOH were added and incubated for 30 min, then the expression was induced with IPTG (end concentration 0.2 mM) for 24 h at 16°C and 200 rpm. The protein was purified in the same way as described for *Tk*MAT.

### Size exclusion chromatography

2 mL of the protein solution (5 mg/mL) were loaded on a gel filtration column (Superdex 200 prep grade beads in an XK 16/70 glass column [GE Healthcare]) equilibrated with 40 mM Tris-HCl, 150 mM NaCl, pH 8.0 and eluted at a flow rate of 1 mL/min. To estimate the native size of the protein the column was calibrated with a mixture of proteins of known size.

### HPLC assay

The activity of the MAT was determined in 0.1 M Tris-HCl, 0.02 M MgCl_2_, 0.2 M KCl, pH 8.0 using varying amounts of l-methionine and ATP (each 10 mM for standard assays). Assays were performed with 0.5 mg *Tk*MAT in 1 mL end volume at 37°C. The enzyme reaction was stopped after 3 min by the addition of 2% (v/v) HClO_4_ and subsequently neutralised with NaOH. Separation of products was achieved by HPLC (Agilent 1100 Series) using a Sphere-Image 5 SCX column, (250 × 4.6 mm). The mobile phase used was as described by Kamarthapu *et al.*[6] using a flow rate of 1.5 mL/min. Standards were dissolved in the enzyme reaction buffer described above. Kinetic parameters were calculated by non-linear fitting using the Origin software.

### Circular dichroism (CD) spectroscopy

CD spectra were measured with a Jasco J-810 Spectropolarimeter. 1 μM enzyme in 3 mL HPLC assay buffer were heated from 20°C to 100°C in 0.1°C steps and the CD spectrum was measured at 224 nm.

### Crystallisation

*Tk*MAT was crystallised using the sitting drop vapour diffusion method. 1 μL of protein solution (10 mg/mL) was mixed with the same volume of a reservoir solution and equilibrated against 500 μL of the same reservoir in sealed Cryschem 24-1 SBS plates. SeMet-labelled protein crystallised as octahedral crystals belonging to the tetragonal space group P43, using a reservoir solution containing 22% (w/v) polyethylene glycol 3350 and 0.2 M sodium citrate at room temperature. A different, rhombic crystal morphology was obtained from a condition containing 50 mM HEPES/NaOH buffer at pH 7.5, 35% pentaerythritol propoxylate, 3% (w/v) of sucrose and 0.2 M KCl.

### Data collection

In order to facilitate cryo-protection, the crystals were dehydrated by addition of an additional 500 μL of PEG 3350 (50%) to the reservoir on the day before harvesting. Crystals were mounted into nylon loops and flash-cooled in liquid nitrogen. Diffraction data sets were collected at the Swiss Light Source (Paul-Scherrer-Institut, Villigen, Switzerland) either at beam line X06DA with a Pilatus 2M detector or at beam line X06SA with a Pilatus 6M detector (Dectris). 360° of data were collected in steps of 0.5° per image. For phase determination by single-wavelength anomalous dispersion (SAD), a data set was collected at the peak wavelength of the selenium K-edge at 0.9796 Å. The octahedrally-shaped crystals of the SeMet derivative diffracted to 3.8 Å resolution. The *C*2 crystal form was used to collect a native data set to a limiting resolution of 2.0 Å.

### Data processing and structure solution

Images were indexed using XDS [29] and scaled with SCALA [30]. The phase was determined using a combination of SAD and molecular replacement using the programs PHASER_MR and PHASER_EP [31]. *Ss*MAT (PDB-ID 4HPV) was used as replacement model. The SAD experiment revealed 64 Se-Atoms in 8 monomers in the asymmetric unit. The structural model was built in COOT [32] and refined using REFMAC 5 [33]. Data collection and refinement statistics are summarised in Table 3.

**Table 3:** Data collection and refinement statistics

**Data set**	**SeMet derivative**	**Native**
**PDB accession code**	--	4L4Q
**Space group**	*P*4_3_	*C*2
**Unit cell parameters**		
**a, b, c [Å]**	121.2, 121.2, 247.1	134.8, 57.8, 236.4
**α, β, γ [°]**	90.0, 90.0, 90.0	90.0, 104.0, 90.0
**Monomers per a.u.**	2	4
**Wavelength [Å]**	0.9796	1.0000
**Resolution [Å]**	50.0 – 3.8 (3.9 – 3.8)^*^	50.0 – 2.0 (2.1 – 2.0)^*^
**Multiplicity**	6.3 (6.4)	4.7 (3.7)
**Completeness [%]**	99.9 (100.0)	99.2 (95.0)
**I/ σ(I)**	9.9 (3.9)	9.1 (1.9)
**No. of unique reflections**	35,046 (5,077)	119,397 (16,072)
***R***_**int**_	0.144 (0.476)	0.101 (0.491)
***R***_**p.i.m.**_	0.062 (0.203)	0.051 (0.273)
***R***_**cryst**_		0.187 (0.295)
***R***_**free**_		0.244 (0.370)
**est. coordinate error [Å]**		0.141
**r.m.s.d. bond lengths [Å]**		0.017
**r.m.s.d. bond angles [°]**		1.941
**average B-factor [Å**^**2**^**]**		41.0

* values in parentheses represent the highest resolution shell.

Figures were generated using PyMOL (Schrödinger LLC), interactions of ligands with active site residues are based on the corresponding structures using the PoseView application [34].

## Abbreviations

ATP: Adenosine triphosphate; CD: Circular dichroism; EcMAT: MAT from *Escherichia coli*; HsMAT: MAT from *Homo sapiens*; IPTG: Isopropyl thiogalactoside; MAT: Methionine adenosyltranferase; MjMAT: MAT from *Methanocaldococcus jannaschii*; SAD: Single wavelength anomalous dispersion; SAM: *S*-adenosylmethionine; SCX: Strong cation exchange; SeMet: Selenomethionine; r.m.s.d: Root-mean-squared displacement; SsMAT: MAT from *Sulfolobus solfataricus*; TkMAT: MAT from *Thermococcus kodakarensis.*

## Competing interests

The authors declare that they have no competing interests.

## Authors' contributions

JSc carried out size exclusion chromatography, crystallisation, data measurement of *Tk*MAT, performed structure-based alignments and helped to draft the manuscript. JSi performed expression and purification of *Tk*MAT, kinetic studies, CD measurements and helped to draft the manuscript. SG solved the phase of the crystals. AE constructed the *tkmat* expression vector. SB established the expression protocol. MR established the HPLC assay, participated in initial activity measurements and drafted the manuscript. OE refined and analysed the *Tk*MAT structure and drafted the manuscript. JNA conceived of the study, participated in its design and coordination and drafted the manuscript. All authors read and approved the final manuscript.

## Supplementary Material

Additional file 1 **Figure S1.** Size exclusion chromatography of *Tk*MAT. **Figure S2**: Thermal denaturation of *Tk*MAT. **Figure S3**: Determination of kinetic parameters for *Tk*MAT. **Figure S4**: Comparison of open and closed MAT structures. **Figure S5**: Surface representation of MAT monomers. Click here for file
