# Supplementary material for: Structural and functional characterisation of the methionine adenosyltransferase from Thermococcus kodakarensis
Source: BMC Struct Biol. 2013 Oct 18;13:22. doi: 10.1186/1472-6807-13-22 (PMC3853416; doi:10.1186/1472-6807-13-22)
Supplement: Additional file 1 — Figure S1. Size exclusion chromatography of TkMAT. Figure S2: Thermal denaturation of TkMAT. Figure S3: Determination of kinetic parameters for TkMAT. Figure S4: Comparison of open and closed MAT structures. Figure S5: Surface representation of MAT monomers. [file 1472-6807-13-22-S1.pdf]

Supplemental information

Structural and functional characterisation of the methionine adenosyltransferase from *Thermococcus kodakarensis*

Julia Schlesier, Jutta Siegrist, Stefan Gerhardt, Annette Erb, Simone Bläsi, Michael Richter, Oliver Einsle, Jennifer N. Andexer

Figure S1: Size exclusion chromatography of *Tk*MAT.
Figure S2: Thermal denaturation of *Tk*MAT.
Figure S3: Determination of kinetic parameters for *Tk*MAT.
Figure S4: Comparison of open and closed MAT structures.
Figure S5: Surface representation of MAT monomers.

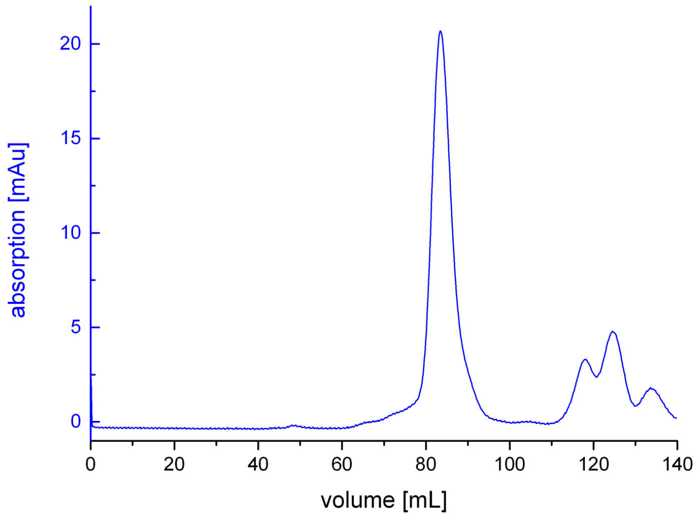

Figure S1: Size exclusion chromatography of *Tk*MAT. *Tk*MAT elutes in a single peak at a volume corresponding to a molecular mass of 81 kDa, clearly showing that the enzyme (monomer 46.8 kDa) is a dimer in solution. For calibration of the column a commercial protein standard mix (HMW calibration kit, GE Healthcare, consisting of ovalbumin [43 kDa], conalbumin [75 kDa], aldolase [158 kDa], ferritin [440 kDa] and thyroglobulin [669 kDa]) plus carboanhydrase (29 kDa) was used resulting in a calibration curve with the equation: $y = 10^{5.33 - 0.041 \cdot x}$

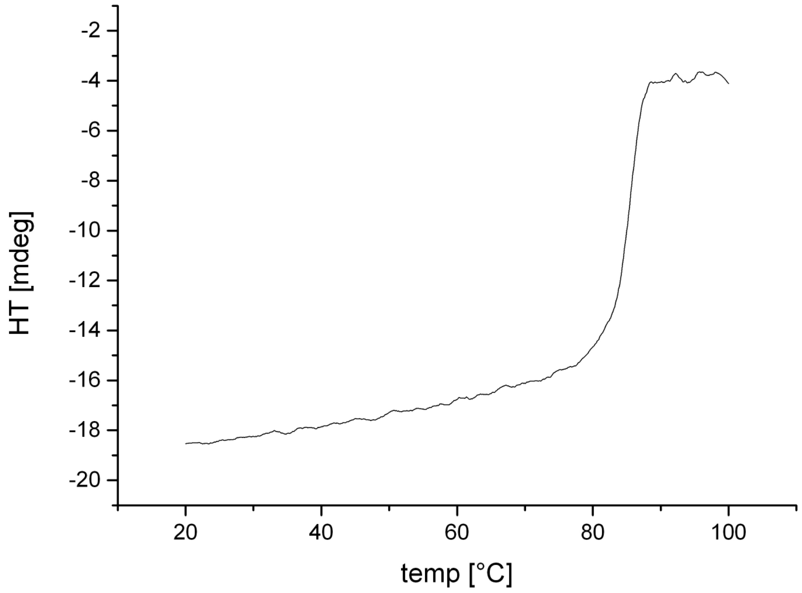

Figure S2: Thermal denaturation of *Tk*MAT. The inflection point of the graph (85 °C) corresponds to T_{50}.

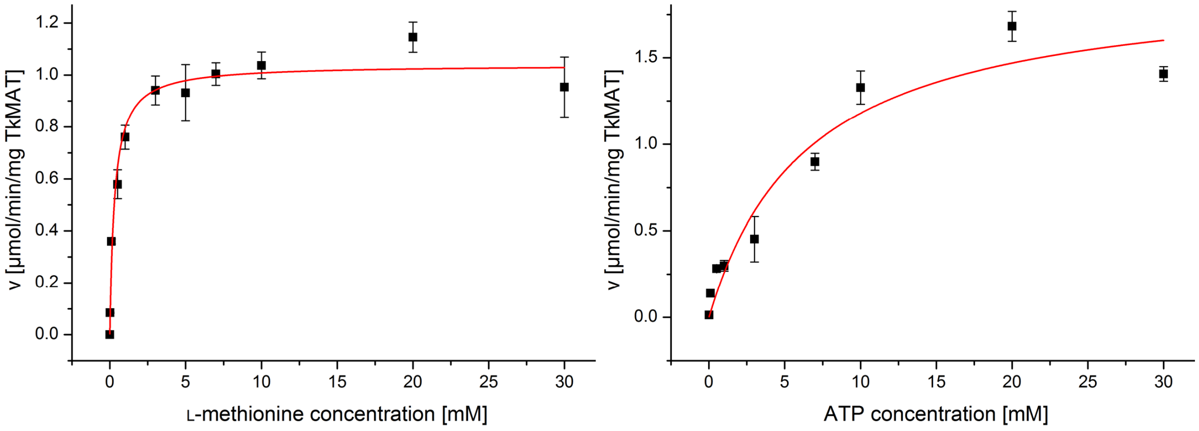

Figure S3: Determination of kinetic parameters for *Tk*MAT. Left: variation of methionine concentration at constant ATP concentration (10 mM); right: variation of ATP concentration at constant methionine concentration (10 mM). Kinetic parameters were determined by non-linear fitting with the Origin software: K_M (Met) = 0.31 ±0.07 mM, K_M (ATP) = 6.54 ±2.49 mM, v_{max} = 1.95 ± 0.26 µmol/min/mg.

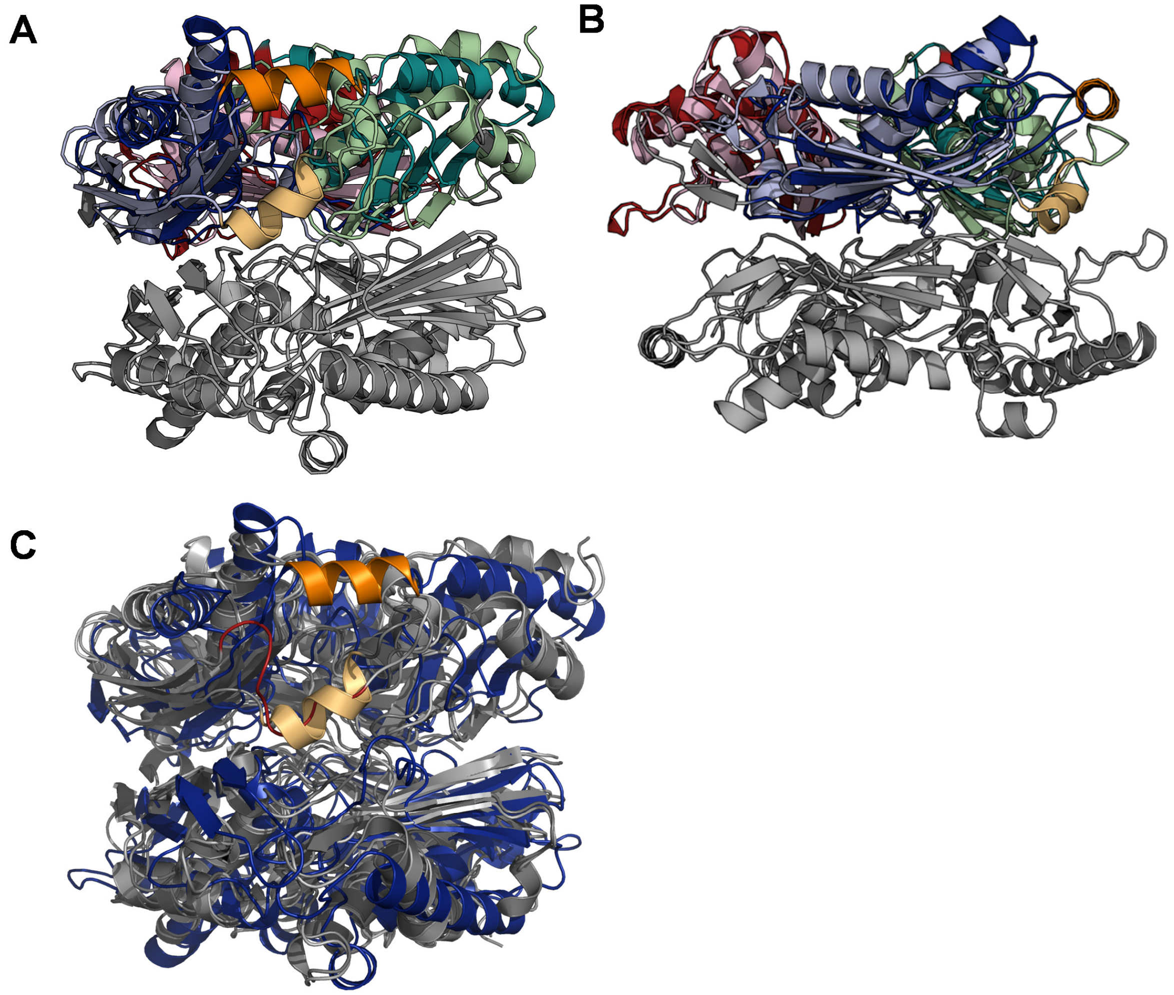

Figure S4: Comparison of open and closed MAT structures. A: overlay (upper subunit) of *Tk*MAT in the open state and *Ec*MAT in the closed state (1RG9). B: side view of A. C: overlay of *Tk*MAT (open, orange helix) and two *Ec*MAT structures: 1RG9 (closed, light orange helix) and 1FUG (quasi-closed/disordered loop, red loop).

A: *Ec*MAT

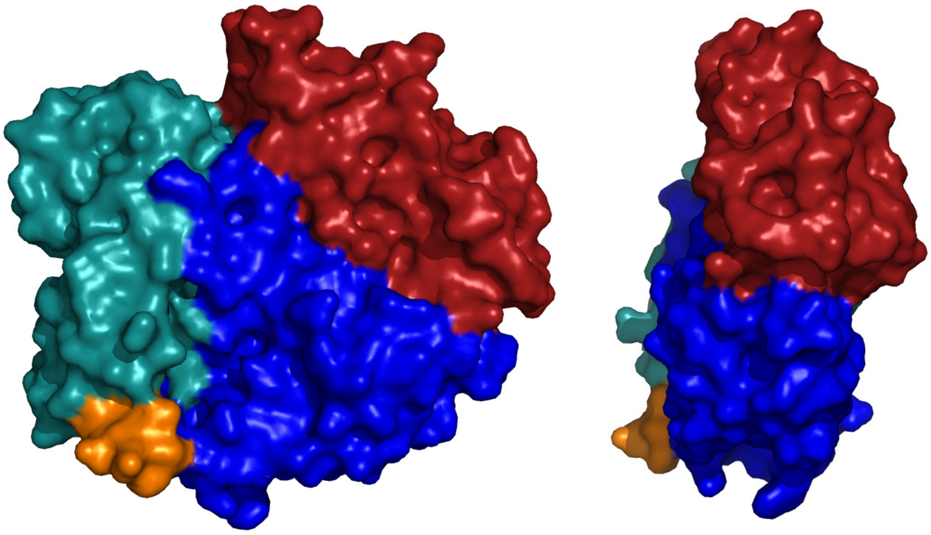

B: *Tk*MAT

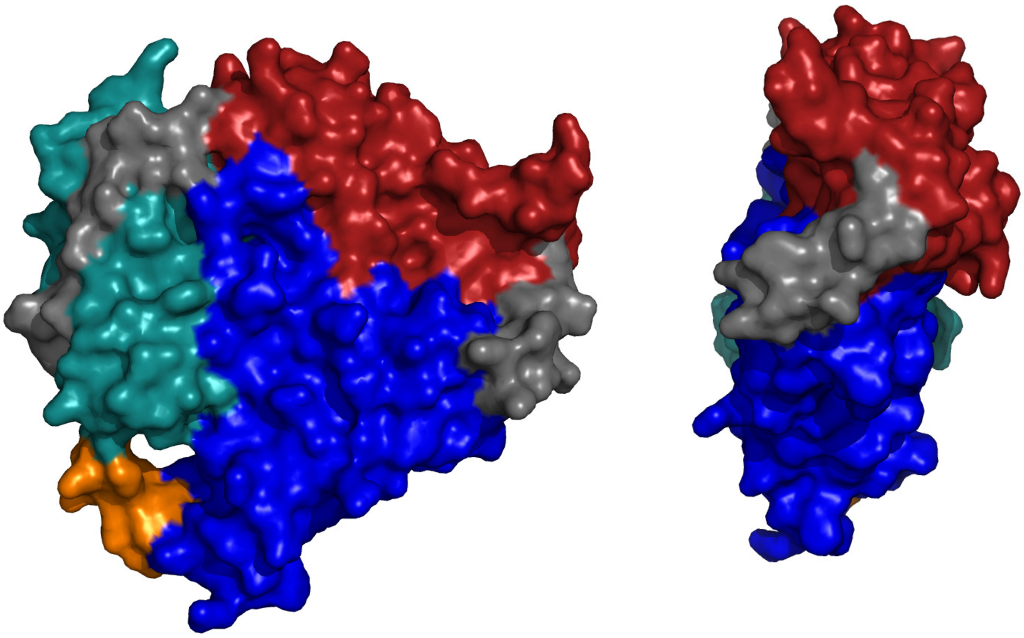

Figure S5: Surface representation of MAT monomers. Left: dimer interface, right: side view. The additional β-strands present in *Tk*MAT are highlighted in grey. The very first β-strand is part of the C-terminal domain (cyan), whereas the second additional element is part of the central domain (blue) and wraps around the loop region of the N-terminal domain (red), where it could be beneficial for the overall stability of the enzyme.
